# Deep brain stimulation in the medial septum attenuates temporal lobe epilepsy via entrainment of hippocampal theta rhythm

**DOI:** 10.1111/cns.13617

**Published:** 2021-01-27

**Authors:** Ying Wang, Yating Shen, Xianhui Cai, Jie Yu, Cong Chen, Bei Tan, Na Tan, Heming Cheng, Xiang Fan, Xiaohua Wu, Jinggen Liu, Shuang Wang, Yi Wang, Zhong Chen

**Affiliations:** ^1^ Key Laboratory of Neuropharmacology and Translational Medicine of Zhejiang Province College of Pharmaceutical Science Zhejiang Chinese Medical University Hangzhou China; ^2^ Institute of Pharmacology and Toxicology College of Pharmaceutical Sciences Zhejiang University Hangzhou China; ^3^ Epilepsy Center School of Medicine Second Affiliated Hospital Zhejiang University Hangzhou China

**Keywords:** cognitive function, deep brain stimulation, epilepsy, medial septum

## Abstract

**Aims:**

Temporal lobe epilepsy (TLE), often associated with cognitive impairment, is one of the most common types of medically refractory epilepsy. Deep brain stimulation (DBS) shows considerable promise for the treatment of TLE. However, the optimal stimulation targets and parameters of DBS to control seizures and related cognitive impairment are still not fully illustrated.

**Methods:**

In the present study, we evaluated the therapeutic potential of DBS in the medial septum (MS) on seizures and cognitive function in mouse acute and chronic epilepsy models.

**Results:**

We found that DBS in the MS alleviated the severity of seizure activities in both kainic acid‐induced acute seizure model and hippocampal‐kindled epilepsy model. DBS showed antiseizure effects with a wide window of effective stimulation frequencies. The antiseizure effects of DBS were mediated by the hippocampal theta rhythm, as atropine, which reversed the DBS‐induced augmentation of the hippocampal theta oscillation, abolished the antiseizure effects of DBS. Further, in the kainic acid‐induced chronic TLE model, DBS in the MS not only reduced spontaneous seizures, but also improved behavioral performance in novel object recognition.

**Conclusion:**

DBS in the MS is a promising approach to attenuate TLE probably through entrainment of the hippocampal theta rhythm, which may be therapeutically significant for refractory TLE treatment.

## Graphical abstract

Here, we inferred that the DBS in the MS may be therapeutically significant for the treatment of refractory TLE. DBS in the MS not only alleviated seizures activities, but also improved behavioral performance in memory tests, via the enhancement of hippocampal theta rhythm.

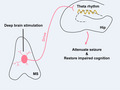

## 1 INTRODUCTION

Despite advances in pharmacotherapy for epilepsy, more than 30% of the patients still have incompletely controlled seizures.^1^, ^2^ Temporal lobe epilepsy (TLE) is one of the most common types of medically refractory epilepsy, often associated with cognitive impairment.^3^, ^4^ In this refractory population, surgery is often considered to be an effective therapeutic choice, but many patients are not good candidates because of multiple seizure foci, foci in eloquent brain regions, or some that cannot be identified.^5^ Poor control of seizures and seizure‐related complications is a heavy burden for patients and society.^6^ Thus, there is a pressing need to develop alternative therapeutic approaches to control intractable TLE.

During recent decades, deep brain stimulation (DBS) has become a new option for refractory epilepsy treatment and accompanied memory impairment with the advantages of reversibility, adjustability, and low risk of complications.^7^, ^8^ Both clinical and animal studies have demonstrated that DBS targeting specific brain areas or fibers, the seizure foci or the key regions of seizure spread, protects against epileptic seizures. For example, DBS in anterior nucleus of thalamus shows promising therapeutic efficacy in the treatment of refractory epilepsy, which has been well investigated in human^9^ and nonhuman primate study.^10^ Meanwhile, vagal nerve stimulation^11^ and responsive neurostimulation^12^ have also been applied for seizure control. Our previous animal studies also demonstrated that DBS at crucial regions, such as the subiculum,^13^, ^14^, ^15^ amygdala,^16^ entorhinal cortex,^17^ inhibits seizures in different epilepsy models. However, the response rates of current targets are relatively low and usually a long time is needed to achieve its anti‐epileptic efficacy. The therapeutic outcome of DBS is largely dependent on its target and stimulation parameter, which may account for much conflicting data in different studies.^18^, ^19^ Currently, the optimal stimulation targets and parameters of DBS to control seizures and related cognitive impairment are still not fully illustrated.

The medial septum (MS), located in basal forebrain, belongs to limbic system regions and has reciprocal pathways with the hippocampus.^20^ MS‐hippocampal projections are critical for hippocampal theta oscillations that mediate learning and memory.^21^ Recently, much attention has been paid to its role in epilepsy.^22^ EEG activity in the MS and hippocampus is highly correlated during seizure activities.^23^ Clinical data demonstrated that TLE patients show abnormal structure and function in the MS.^24^, ^25^ There are also neuronal loss and reorganization in the MS in chronic epilepsy model.^26^ Using optogenetics, we recently revealed the precise role of three types of MS neurons in epilepsy and found that direct MS‐hippocampal cholinergic circuit is intrinsically antiseizure.^27^ Those results indicated that the MS may be a promising target for DBS to inhibit seizure and restore associated memory impairment. However, whether the MS is a promising target of DBS suitable for general epilepsy models is still unclear. Thus, in the present study, we aimed to investigate the therapeutic potential of DBS in the MS in different types of epilepsy models.

## 2 METHODS

### 2.1 Animals

C57BL/6 mice (male, 25–30 g, 2–4 months old) were used in this study. They were group‐maintained prior to surgery in cages with a 12‐h light/dark cycle (lights on from 8:00 to 20:00). They were individually housed after surgery. The use and care of the mice were approved by the Zhejiang University Animal Experimentation Committee and were in complete compliance with the National Institutes of Health Guide for the Care and Use of Laboratory Animals.

### 2.2 Kainic acid (KA)‐induced acute seizure model

Mice were anesthetized with sodium pentobarbital (50 mg/kg, i.p.). A homemade cannula electrode was stereotaxically implanted into right dorsal hippocampus (AP: −2.0 mm; ML: −1.3 mm; V: −1.6 mm), and a bipolar electrode (each 0.125 mm in diameter, 0.5 mm tip distance, Cat No. 791500, A‐M Systems) was stereotaxically implanted into the MS (AP: +1.0 mm; ML: 0.0 mm; V: −4.0 mm) based on the mouse brain atlas.^28^ After 7 days of recovery from surgery, KA (0.25 μg in 0.5 μL saline) was injected into the hippocampus through cannula over 2 min with a 1‐μL microsyringe. EEGs were recorded and analyzed using a Neuroscan system (Compumedics, Australia). As we reported previously,^29^ seizure severity was classified according to the modified Racine scale.^30^ Status epilepticus (SE) onset was behaviorally characterized as our previous study.^31^ Stages 4–6 were considered to be as generalized seizures (GS). We recorded the EEG for 1.5 h after KA injection and measured the seizure stage, latency to different stages, number of GS, and incidence of GS or SE.

To investigate the effect of DBS in the MS on the KA‐induced acute seizure model, mice were randomly divided into five groups, which received DBS immediately after KA injection. DBS was composed of monophasic square‐wave pulses, 100 μA and 0.1 ms per pulse, at the frequencies of 1, 5, 20, or 100 Hz, respectively. DBS was delivered by a constant current stimulator (SEN‐7203, SS‐202J; Nihon Kohden). The mice in sham group were left in the chamber and connected to the apparatus but no DBS was delivered.

The background EEG in the hippocampus was analyzed off‐line by software package in LabChart 7 and the details for spectral analyses were same as our previous study.^32^ To minimize individual variation, the normalized power ratio in each mouse was calculated through post power (start of DBS) divided by pre power (baseline). For post power, we only analyze the hippocampal background EEG before seizure onset and did not include any non‐seizure epileptic spike, since those spikes may accompany high power. The percent of total power was calculated through the power of certain oscillation divided by the total power.

### 2.3 Hippocampal‐kindled epilepsy model

Under sodium pentobarbital anesthesia (50 mg/kg, i.p.), bipolar electrodes were stereotaxically implanted into the ventral hippocampus (AP: −2.9 mm; ML: −3.1 mm; V: −3.1 mm) and the MS (AP: +1.0 mm; ML: 0.0 mm; V: −4.0 mm). After recovery, mice were performed kindling acquisition as our previous studies.^33^, ^34^ Briefly, all mice received 10 kindling stimulations (400 μA, 20 Hz, 2‐s trains, 1‐ms monophasic square‐wave pulses) daily with 30‐min intervals. Behavioral seizure severity was classified according to the Racine scale, and in this model, mice usually showed the seizure stage 1–5. Stages 1–3 were considered to be focal seizures and stages 4–5 as GSs. When animals had three consecutive seizure stages 5, they were regarded as fully kindled.

To investigate the effect of DBS in the MS on hippocampal‐kindled epilepsy model, fully kindled mice were randomly divided into five groups. The kindled mice in sham group only received kindling stimulation without any DBS, while kindled mice in other four DBS groups received continuous 15‐min DBS treatment at frequencies of 1, 5, 20, or 100 Hz immediately after kindling stimulation. To achieve reliable results and avoid accidental event, the mice in all groups received 3‐time DBS treatments with 1‐h interval and the data were averaged for analysis.

### 2.4 KA‐induced chronic epilepsy model

Mice were anesthetized with isoflurane (5% induction and 1.5% maintenance) and KA (0.25 μg in 0.5 μL saline) was stereotaxically injected into right dorsal hippocampus (AP: −2.0 mm; ML: −1.3 mm; V: −1.6 mm) over 2 min with a 1‐μL microsyringe. Two months after injection, survived mice were anesthetized with isoflurane and bipolar electrodes (each 0.125 mm in diameter, 0.5 mm tip distance, Cat No. 791500, A‐M Systems) were implanted in the right ventral hippocampus (AP: −2.9 mm; ML: −3.1 mm; V: −3.1 mm) for EEG recording and the MS (AP: +1.0 mm; ML: 0.0 mm; V: −4.0 mm) for DBS.

After 7‐day recovery from surgery, EEG was continuously recorded in freely moving mice by PowerLab system (AD Instruments) with 8 h/day for 7 days as baseline (Pre‐DBS). The mice with detectable paroxysmal discharges received 15‐min DBS (monophasic square‐wave pulses, 5 Hz, 0.1 ms per pulse, 100 μA) at the same time every day for continuative 7 days (DBS). EEG was recorded additional 7 days after DBS treatment (Post‐DBS). Paroxysmal discharges were defined as regular spike clusters with a duration >10 s, spike frequency >2 Hz, and amplitude at least three times of the baseline EEG, as our previous study.^35^ The “Normalized number of paroxysmal discharges” means the absolute number of paroxysmal discharges for every day was standardized to the mean number of paroxysmal discharges for the 7‐day baseline period. The “Normalized duration of paroxysmal discharges” means the absolute duration of paroxysmal discharges for every day was standardized to the mean duration of paroxysmal discharges for the 7‐day baseline period.

### 2.5 Learning and memory tests

The learning and memory tests, including novel object recognition and object‐location memory tests, were performed in the same animals before and after DBS treatment. The tests for pre‐DBS were performed two months after KA injection when recurrent spontaneous seizures are stable (before DBS treatment). The tests for post‐DBS were performed on the day next to post‐DBS period. The time interval between pre‐DBS and post‐DBS was about one month. The details for the procedures of novel object recognition and object‐location memory tests were same as our previous study.^32^ The exploration time spent on each of the familiar (F) object and the displaced (D) object or the new (N) object was recorded manually. The discrimination index was calculated by (D‐F/D + F) ×100% or (N‐F/N + F) ×100% for intergroup comparisons.

### 2.6 Histology

Mice were deeply anesthetized with pentobarbital (100 mg/kg, i.p.) and perfused transcardially with saline followed by 4% paraformaldehyde in 0.1 M phosphate buffer. We removed the brains and post‐fixed them in 4% phosphate‐buffered paraformaldehyde at 4°C overnight and cryoprotected the brains with 30% (w/v) sucrose. Coronal 30‐μm sections were cut with a sliding freezing microtome (Leica) to identify the location of electrodes and cannula.

### 2.7 Statistics

Data are presented as the mean ±s.e.m. Number of experimental replicates (n) is indicated in each figure legend. Statistical comparisons were performed using Prism (version 7.0) with appropriate statistical methods as indicated in figure legends. Statistical tests were applied after testing for normal distribution (Shapiro‐Wilk test). Non‐parametric tests were applied for the data not complying with the normal distribution. Parametric tests were applied for the data complying the normal distribution. Moreover, correction tests were used for multiple comparisons. No statistical methods were used to pre‐determine sample size, or to randomize. A two‐tailed *P* value <0.05 was considered statistically significant.

## 3 RESULTS

### 3.1 DBS in the MS alleviates seizure activities in epilepsy models

**Figure 1:**
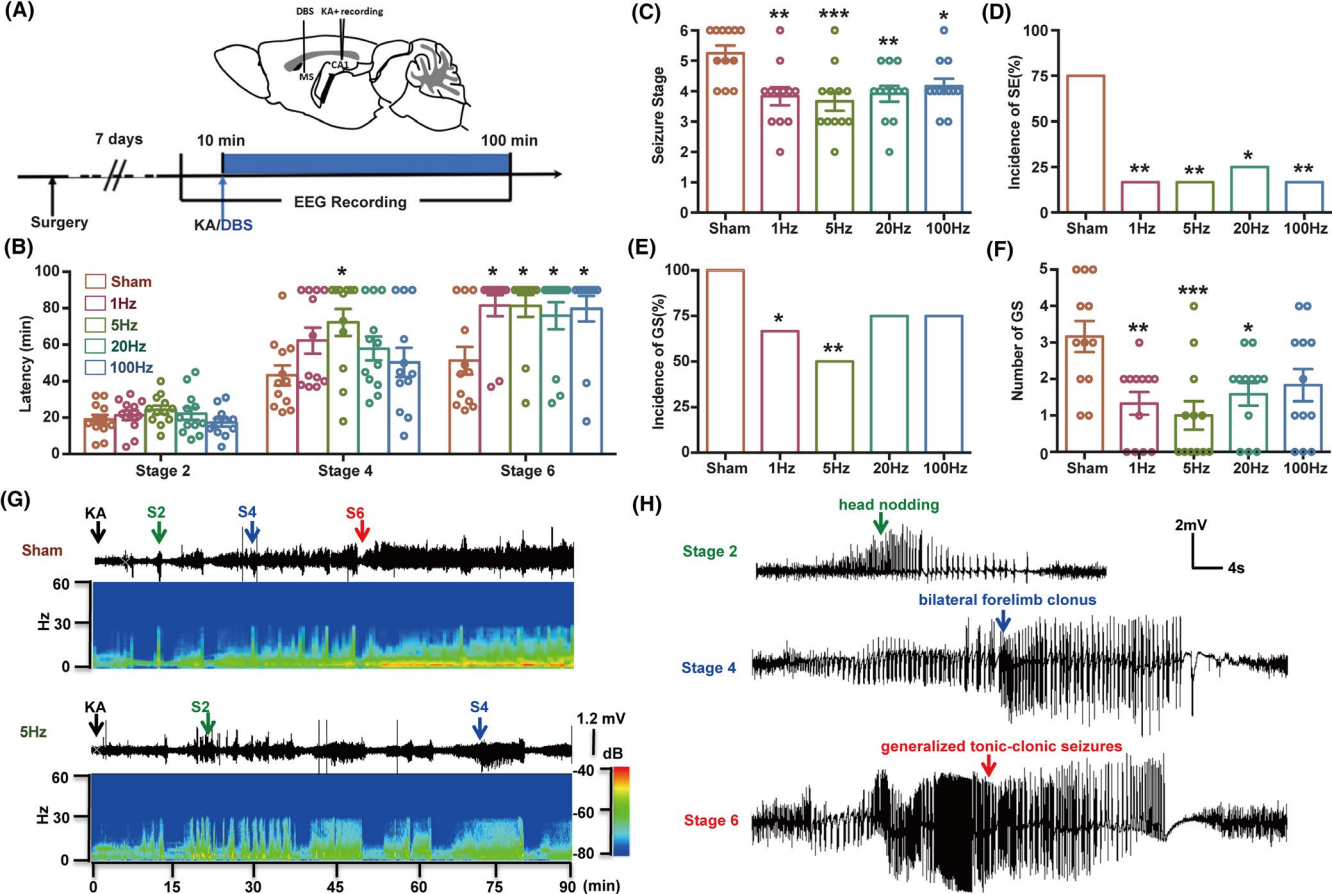
DBS in the MS alleviates seizure activities in KA‐induced acute seizure model. (A) Experiment scheme diagram of the KA‐induced seizure model and DBS modulation. DBS was delivered continuously after KA injection. (B‐F) The effects of DBS on the latency to the different seizure stage (B), seizure stage (C), incidence of SE (D), incidence of GS (E), and number of GS (F) during 1.5‐h observation period in KA‐induced acute seizure model. (n = 12 for each group. *P < 0.05, **P < 0.01, ***P < 0.001 compared with Sham group, non‐parametric Kruskal‐Wallis test followed by post hoc Dunn's tests was used for B, one‐way ANOVA followed by post hoc Dunnett tests was used for C and F, chi‐square test was used for D and E). (G‐H) Representative power spectrogram (G) and EEGs (H) recorded from the dorsal hippocampus. The arrows indicate the time of KA injection and the first onset time of seizure stage 2, 4, or 6

First, we tested the effect of DBS in the MS on KA‐induced acute seizure model. We implanted a cannula electrode into right dorsal hippocampus of mice to allow administration of KA and EEG recording (Figure 1A). We found that continuous DBS at all the frequencies of 1, 5, 20, or 100 Hz, delivered immediately after KA injection, delayed the latency to seizure stage 6. DBS at the frequency of 5 Hz also delayed the latency to seizure stage 4, while DBS at all frequencies did not change the latency to seizure stage 2 (Figure 1B), indicating that DBS in the MS may interfere seizure spread from focal seizure to GS. Meantime, DBS at all frequencies also lowered the seizure stage (Figure 1C) and incidence of SE (Figure 1D) during 1.5‐h observation period after KA treatment. DBS at the frequencies of 1 and 5 Hz further lowered the incidence of GS (Figure 1E) and reduced the number of GS (Figure 1F, Sham group: 3.2 ± 0.5; 1 Hz group: 1.4 ± 0.3; 5 Hz group: 0.9 ± 0.4; 20 Hz group: 1.6 ± 0.3; 100 Hz group: 1.9 ± 0.5). Typical EEGs during seizure are shown in Figure 1G‐H. Moreover, as intermittent stimulation might be beneficial for preventing unwanted side effects and preserving battery life in the clinic, we also tested the effect of intermittent DBS in the MS on KA‐induced acute model. We found that intermittent DBS in the MS was less effective than continuous DBS (Figure S1). These results indicated that DBS in the MS alleviates seizure activities in KA‐induced acute model with wide window of effective stimulation frequencies.

**Figure 2:**
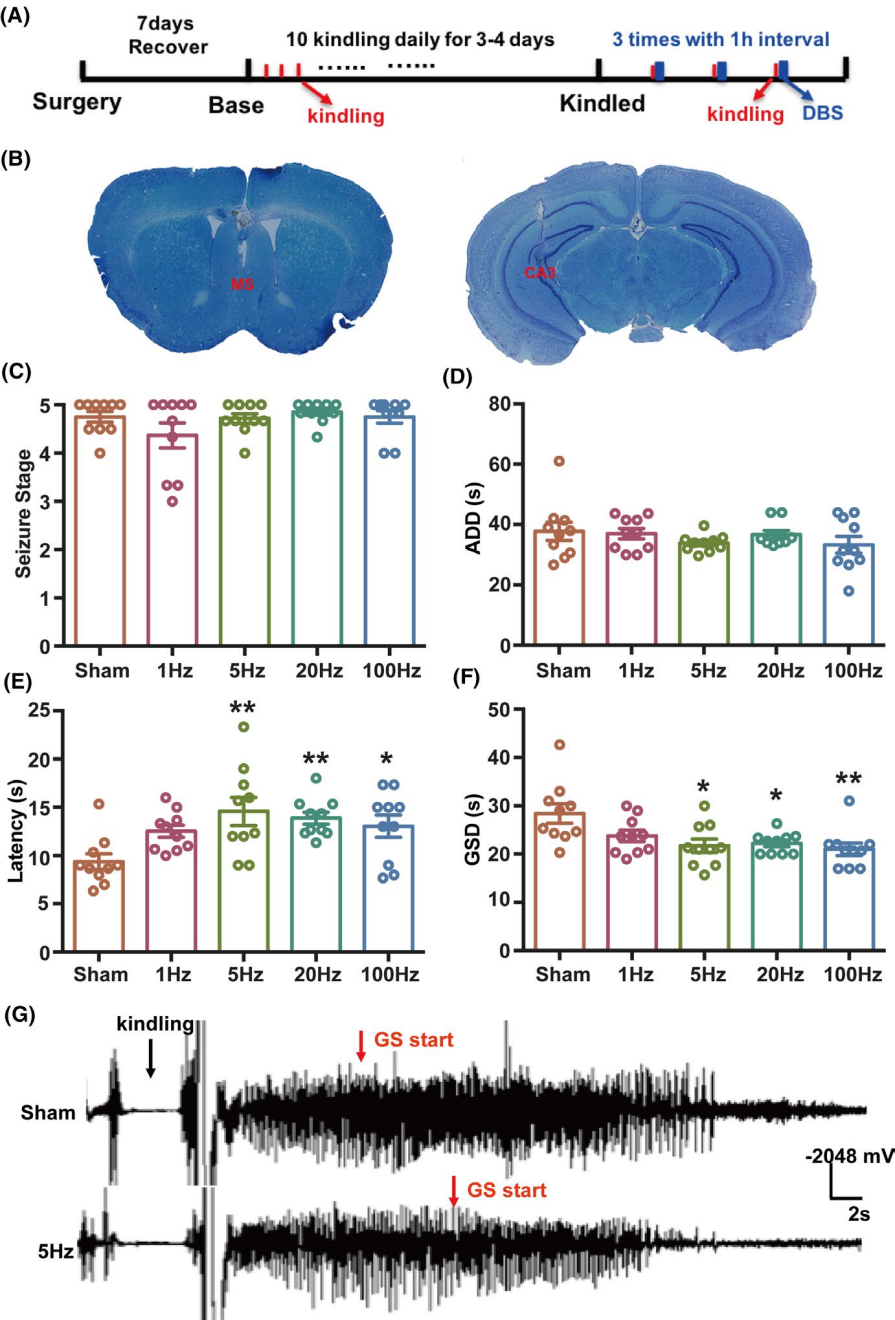
DBS in the MS alleviates seizure activities in hippocampal‐kindled epilepsy model. (A) Experiment scheme diagram of the hippocampal‐kindled seizure model and DBS modulation. DBS was delivered continuously with 15‐min duration immediately after kindling stimulation. (B) Toluidine blue staining for the location of the electrodes in the MS and hippocampus. (C‐F) Seizure stage (C), ADD (D), latency to GS (E), and GSD (F) in hippocampal‐kindled epilepsy model with DBS modulation. (n = 10 for each group. *P < 0.05, **P < 0.01 compared with Sham group, one‐way ANOVA followed by post hoc Dunnett tests was used for E, non‐parametric Kruskal‐Wallis test followed by post hoc Dunn's tests was used for F). (G) Representative EEGs recorded from the dorsal hippocampus

Then, we tested whether DBS in the MS could suppress seizure activity in hippocampal‐kindled epilepsy model, which clinically resembles complex partial seizures with GS.^36^ DBS at the frequencies of 1, 5, 20, or 100 Hz with 15‐min duration was delivered immediately after kindling stimulation (Figure 2A,B) to mimic close‐loop stimulation pattern.^37^ Although DBS did not change seizure stage (Figure 2C) and after‐discharge duration (ADD, Figure 2D), DBS at the frequencies of 5, 20, or 100 Hz extended the latency to GS (Figure 2E, Sham group: 9.4 ± 0.8 s; 5 Hz group: 14.6 ± 1.4 s; 20 Hz group: 13.9 ± 0.6 s; 100 Hz group: 13.0 ± 1.2 s) and shortened GS duration (GSD, Figure 2F, Sham group: 28.4 ± 2.0 s; 5 Hz group: 21.7 ± 1.4 s; 20 Hz group: 22.2 ± 0.7 s; 100 Hz group: 21.0 ± 1.3 s), suggesting that DBS in the MS may retard seizure spread from focal seizure to GS. Typical EEGs during seizure are shown in Figure 2G. Similar as that in acute KA‐induced seizure model, results in hippocampal‐kindled seizure model indicate a wide window of effective stimulation frequency for the DBS intervention in the MS. Meanwhile, to exclude the possibility that DBS‐induced lesion of the MS would block the generalization of seizures, we electrically damaged the MS (Figure S2A,B) and found that it significantly promoted seizure development in kindling model (Figure S2C‐E). Above results indicated that DBS in the MS significantly alleviates seizures in both KA‐induced acute seizure model and hippocampal‐kindled epilepsy model with wide window of effective stimulation frequencies.

### 3.2 Hippocampal theta rhythm is involved in the antiseizure effect of DBS

**Figure 3:**
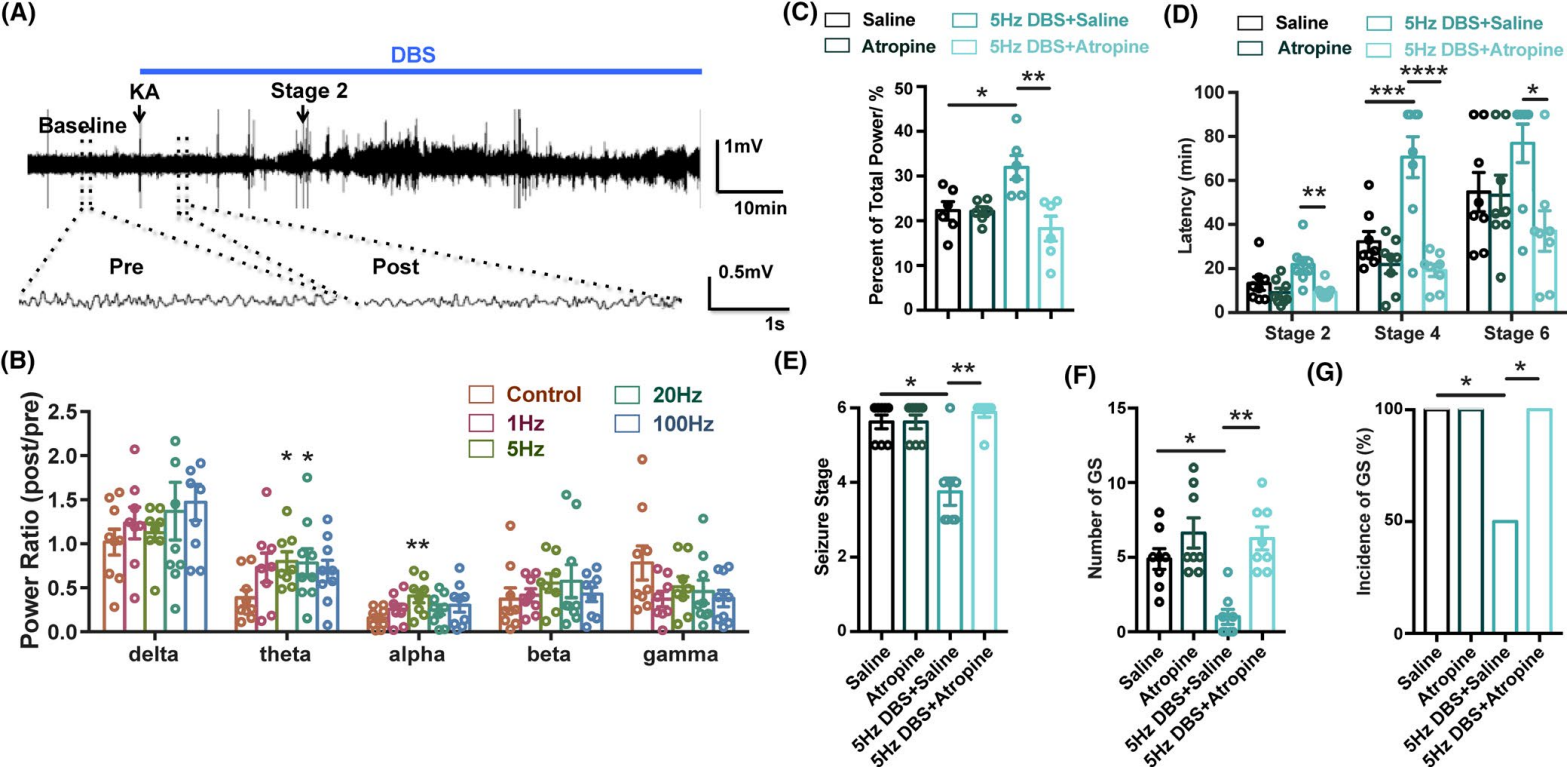
Hippocampal theta rhythm mediates the antiseizure effects of DBS. (A) Experiment scheme for EEG recording and analysis in KA‐induced acute seizure model. (B) The effects of DBS on the spectrum analysis of hippocampal EEG rhythm (n = 8 for each group, *P < 0.05, **P < 0.01 compared with Sham group, one‐way ANOVA followed by post hoc Dunnett tests). To minimize individual variation, the normalized power ratio in each mouse was calculated through post power (start of DBS) divided by pre power (baseline). (C) Atropine abolished the DBS‐induced increase of theta rhythm (n = 6 for each group, *P < 0.05, **P < 0.01, one‐way ANOVA followed by post hoc Bonferroni's test). (D‐G) The effects of DBS on the latency to the different seizure stage (D), seizure stage (E), number of GS (F), and incidence of GS (G) in 1.5‐h observation period in KA‐induced acute seizure model, in the presence of atropine. Atropine was intraperitoneal injected before KA injection, DBS was delivered continuously after KA injection. (n = 8 for each group, *P < 0.05, **P < 0.01, ***P < 0.001, ****P < 0.0001, one‐way ANOVA followed by post hoc Bonferroni's test was used for D, non‐parametric Kruskal‐Wallis test followed by post hoc Dunn's tests was used for E, F, chi‐square test was used for G)

Moreover, we tried to see whether the DBS in the MS has an effect on hippocampal neural activities. Analysis of hippocampal background EEG before seizure onset in KA‐induced acute seizure model showed that DBS at the frequencies of 5 and 20 Hz significantly increased the ratio power of theta oscillation (Figure 3A,B). We did not analyze the hippocampal EEG during seizures, because synchronized high‐amplitude after‐discharge may interfere the modulation effect of DBS on hippocampal EEG. Hippocampal theta band power was reported to be antiseizure,^38^ and we further investigated whether the increased theta power contributes to the antiseizure effect of DBS. As hippocampal theta oscillation is sensitive to MS cholinergic signaling,^39^, ^40^ we found that intraperitoneal injection of atropine before KA injection reversed DBS‐induced augmentation of the hippocampal theta oscillation (Figure 3C) and completely abolished the antiseizure effect of 5‐Hz DBS (Figure 3D‐G). These data suggested that the antiseizure effects of DBS in the MS are probably mediated by the increased hippocampal theta rhythm.

### 3.3 DBS in the MS attenuates chronic seizures and partially restores the impaired cognitive function

**Figure 4:**
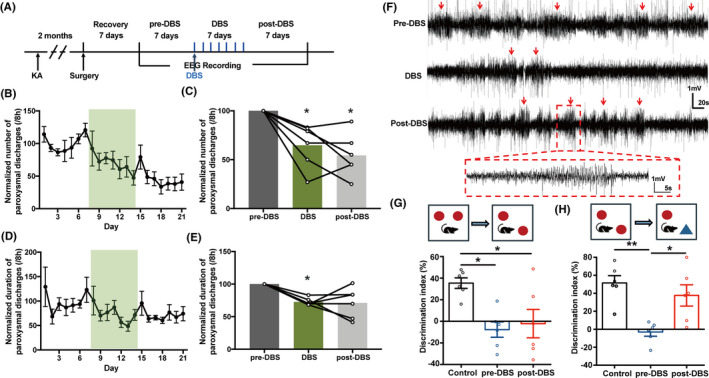
DBS in the MS alleviates seizure activities and partially restores the impaired cognitive function in KA‐induced chronic epileptic mice. (A) Experiment scheme diagram of the KA‐induced chronic seizure model and DBS modulation. (B‐E) To minimize individual variation, the data for each mouse were normalized to the average value of the 7‐days pre‐DBS. The effects of the DBS in the MS on the normalized number of paroxysmal discharges (B, C) and the normalized duration of paroxysmal discharges (D, E) in KA‐induced chronic epilepsy model (n = 6. *P < 0.05 compared with pre‐DBS, Wilcoxon matched‐pairs signed‐rank test). (F) Representative paroxysmal discharges in pre‐DBS, DBS, and post‐DBS periods. An enlarged paroxysmal discharge was shown in the box. (G) The effects of DBS in the MS on the performance of KA‐induced chronic epileptic mice in an object‐location task (n = 6. *P < 0.05, one‐way ANOVA followed by post hoc Bonferroni's test). (H) The effects of DBS in the MS on the performance of KA‐induced chronic epileptic mice in a novel object recognition task (n = 6 for each group. *P < 0.05, **P < 0.01, one‐way ANOVA followed by post hoc Bonferroni's test)

Further, to test whether the antiseizure effect of DBS in the MS is also applied to the chronic TLE model, we used intrahippocampal KA chronic epilepsy model, which resembles the typical patterns of hippocampal pathology in human TLE and develops drug resistance.^41^ Two months after KA injection, spontaneous recurrent paroxysmal discharges were recorded in ventral hippocampus. In order to determine the effect of DBS in the MS on recurrent spontaneous seizures, we analyzed the number and duration of paroxysmal discharges before and after 5‐Hz DBS (Figure 4A). The number and duration of paroxysmal discharges were stable over the 7‐day baseline period. DBS at the frequency of 5 Hz in the MS significantly reduced the normalized number (Figure 4B,C, DBS group: 64.67 ± 9.095%; post‐DBS group: 54.33 ± 8.939%) and duration of paroxysmal discharges during the treatment period (Figure 4D,E, DBS group: 72.65 ± 2.381%; post‐DBS group: 71.17 ± 9.49%). Typical EEGs are shown in Figure 4F. In addition, sham DBS in the MS did not change the severity of seizure in KA chronic model (Figure S3). Interestingly, the antiseizure effect of DBS was long‐term and persistent even after DBS treatment period, which might result from the synaptic plasticity induced by DBS.^42^

Cognitive impairment is a common complication of chronic epilepsy.^4^ As hippocampal theta rhythm, which can be enhanced by DBS, was reported to participate in learning and memory,^21^ we investigated the effects of DBS in the MS on the impaired cognition in object‐location task and novel object recognition. Compared with the normal mice, chronic epileptic mice spent less time around the location‐changed object (Figure 4G) and novel object (Figure 4H), indicating impaired cognition. We found that 5‐Hz DBS in the MS for one week improved the performance in novel object recognition task (Figure 4G), while not in object‐location task (Figure 4H). These results indicated that 5‐Hz DBS in the MS not only alleviates seizure activities, but also partially restores the impaired cognitive function in KA‐induced chronic epileptic mice.

## 4 DISCUSSION

DBS is emerging as an alternative approach for refractory epilepsy in the past two decades, although the research about optimal targets and stimulation parameters is still ongoing. Previous studies mainly focused on the target that could control seizures, with less attention on cognitive impairment. In the present study, we revealed critical role of the MS in TLE and accompanied cognitive impairment. Firstly, we revealed that DBS in the MS at wide window of effective stimulation frequencies alleviated seizure activities in both KA‐induced and hippocampal‐kindled epilepsy models. Further, in the chronic TLE model, DBS in the MS not only alleviated spontaneous recurrent seizures, but also partially restored the memory impairment. Combined with previous study that the theta frequency stimulation of the MS would increase seizure thresholds in pilocarpine‐induced rats epilepsy model,^43^, ^44^ it is indicated that the MS may be a promising target of DBS for controlling both seizure and memory impairment in TLE.

Importantly, DBS in the MS achieved antiseizure effects with wide window of effective stimulation frequencies. We found that DBS in the MS at the frequencies of 1, 5, 20, or 100 Hz, all attenuated seizure activities in KA‐induced or hippocampal‐kindled models. Particularly, DBS in the MS at all stimulation frequencies could effectively increase the latency to GS and shorten GSD, indicating that the MS may be mainly involved in seizure spread from focal seizure to GS. In previous studies, DBS in the MS with different frequencies was also reported to have therapeutic effects on epilepsy and related function deficit. For example, Gurkoff et al. reported that 7.7‐Hz DBS in the MS increases seizure threshold in pilocarpine‐induced seizure model.^43^ Jeong et al. reported that 60‐Hz DBS in the MS achieves memory improvement in the Morris water maze.^45^ The therapeutic outcome of DBS is largely dependent on stimulation parameters,^19^ which may undergo a fine‐tuning depending on clinical needs. Among them, frequency is the parameter that leads to more complex scenarios. For example, high‐frequency stimulation (HFS, usually ≥100 Hz) has shown to be effective in improving most parkinsonian signs in the clinic, but long‐term efficacy for axial signs is not always retained, or even detrimental. While low‐frequency stimulation (LFS) has been proved to be clinically useful in lessening the detrimental effects of HFS.^46^ Moreover, prolonged HFS may interrupt the normal function of the stimulated targets and cause tissue damage.^47^ Therefore, a large window of stimulation frequency of DBS would be beneficial in avoiding the adverse effects induced by HFS and improve the overall clinical response. Some brain regions, such as anterior nucleus of thalamus^9^, ^32^ and entorhinal cortex,^17^ have been considered as most attractive targets for DBS to control seizure in TLE. However, the ranges of effective DBS frequency for these regions are usually narrow, and different frequencies used may result in controversial results,^32^, ^48^ which would restrict their clinic application. Here, we demonstrated that the MS might be an important target of DBS with the advantage of wide window of effective stimulation frequencies, which may contribute to clinical efficacy and safety of DBS treatment for epilepsy.

Further, spectrum analysis showed that LFS in the MS significantly increased theta band power in the hippocampus. Hippocampal theta rhythm usually appears during exploration search,^49^ and it can be disrupted or abolished in epilepsy.^50^, ^51^ In fact, in KA‐induced acute seizure model, there is a significant decrease in theta rhythm of background EEG before seizure onset. As increasing the hippocampal theta rhythm by the injection of carbachol into the MS could reduce hippocampal epileptiform spiking,^52^ LFS‐induced augmentation of the hippocampal theta rhythm in our study may be correlated with antiseizure effects. We further used atropine to disrupt theta rhythm as previous study^39^ and found that atropine also abolished the antiseizure effect of LFS. This indicated that hippocampal theta rhythm might mediate the antiseizure effect of LFS in the MS. Certainly, we cannot deny that atropine might be related to other mechanisms, except the theta rhythm, underlying the antiseizure effect of DBS, which deserve further research. The MS is extensively considered to be the pacemaker of the hippocampal theta rhythm and disturbances of septo‐hippocampal theta oscillations were generally reported in the epileptic brain,^53^ which were thought to be closely related to its indirect “cholinergic neuron‐GABAergic neuron” circuit.^40^ LFS may enhance local cholinergic microcircuit or long‐range projecting GABAergic neuron to amplify the hippocampal theta rhythm and thus produced antiseizure effects. Moreover, we also found that HFS in the MS could exert antiseizure effect in acute seizure models, but could not drive hippocampal theta rhythm, which indicated that the other mechanisms may be involved in antiseizure effect of HFS in the MS. As HFS is generally inhibitory, we hypothesized that the antiseizure effect of HFS at MS might be mediated by the inhibition of pro‐seizure MS glutamatergic neurons.^27^ Moreover, apart from the modulation of neural activities, the antiseizure effect of DBS might also be related with the neuroinflammation and microglia polarization, which has been implicated in epilepsy.^54^, ^55^ Although electrical stimulation is not cell specific compared with optogenetic intervention, DBS may be easier to have a clinical translation for epilepsy treatment. Certainly, a critical concern for contemporary neuroscience is to establish whether the hypothesis of theta rhythm in rodents can be translated to humans. Although theta rhythm in humans may be less detectable than rodents, careful observation could identify theta and correlate it to cognitive tasks in humans.^56^, ^57^, ^58^ Moreover, theta rhythm was reported to encode self‐motion and spatial navigation in patients with TLE.^59^ Therefore, it is valuable to translate the theta rhythm‐related mechanism underlying the antiseizure effect of LFS in rodents to patients with epilepsy, which will offer additional insight into its clinic therapy.

Cognitive impairment is a common complication of TLE with hippocampal sclerosis, as the hippocampus is critical for the memory function. In the KA‐induced chronic TLE model, we found that epileptic mice showed impaired cognition in both location‐changed object and novel object. Interestingly, 5‐Hz LFS in the MS improved the performance in novel object recognition task, but not in object‐location task. The possible reasons may be (a) LFS‐induced augmentation of the hippocampal theta rhythm is sensitive to intrinsic non‐spatial memory, like novel object recognition task.^60^ (b) As object‐location memory and novel object memory appear to rely on unique brain structures,^61^ LFS‐induced functional change of neural circuit may be much more closely related to brain regions involved in novel object memory. (c) Difference of stimulation paradigm might be needed for different types of memory tests. The positive effect of LFS in the MS on spatial cognition might be limited within a narrow time window^45^, ^62^ and possibly vanished after LFS withdrawn. Although the underlying mechanism remains to be investigated, our results at least indicated that 5‐Hz LFS partially restores the impaired cognitive function in KA‐induced chronic epileptic mice.

In conclusion, our results demonstrated that the MS may be a unique target of DBS with the advantage of wide window stimulation frequencies to control seizures and restore impaired cognitive function in epilepsy, probably through entrainment of the hippocampal theta rhythm. This may be of great therapeutic significance for clinical treatment of refractory TLE with DBS.

## CONFLICT OF INTEREST

None of the authors has any conflict of interest to disclose.

## Supporting information

Figure S1‐S3

## Data Availability

The data that support the findings of this study are available from the corresponding author upon reasonable request.
