# Supplementary material for: Deep brain stimulation in the medial septum attenuates temporal lobe epilepsy via entrainment of hippocampal theta rhythm
Source: CNS Neurosci Ther. 2021 Jan 27;27(5):577–86. doi: 10.1111/cns.13617 (PMC8025637; doi:10.1111/cns.13617)
Supplement: Supplementary file 1 — Figure S1‐S3 [file CNS-27-577-s001.pdf]

Supplementary information

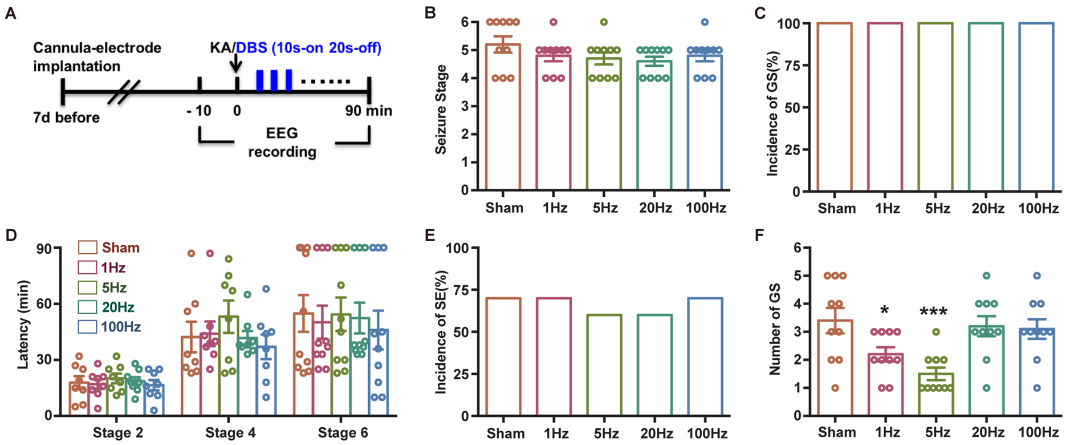

Figure S1 The effect of intermittent DBS in the MS on KA-induced acute seizure model. (A) Schematic diagram of delivery of intermittent DBS (10s "on" state and 20s "off" state) in the MS. **(B-F)** The effects of intermittent DBS on the latency to the different seizure stage **(B)**, seizure stage **(C)**, incidence of SE **(D)**, incidence of GS **(E)**, and number of GS **(F)** during 1.5-h observation period in KA-induced acute seizure model. (n=10 for each group. *$p<0.05$, ***$p<0.001$ compared with Sham group, one-way ANOVA followed by post hoc Dunnett test was used for F).

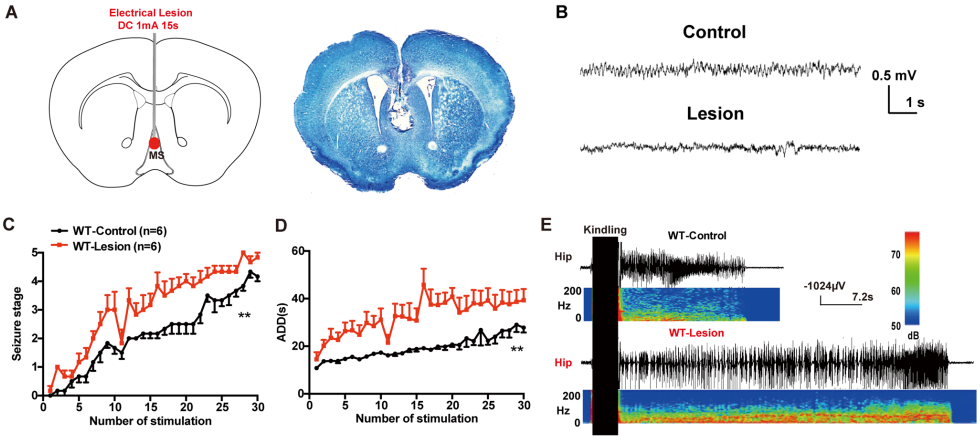

Figure S2 Electrical lesion of the MS accelerates seizure development in hippocampal kindling model. (A) Left, schematic diagram of delivery of electrical lesion (by a 1-mA direct current with 15-s duration) in the MS; Right, representative toluidine blue staining in a coronal section of the WT-Lesion mice. **(B)** The representative EEGs of the MS in a WT-Control mouse and a WT-lesion mouse. **(C, D)** The effects of electrical lesion of the MS on the development of seizure stage **(C)** and ADD **(D)** in hippocampal kindling model; **$p<0.01$ compared with WT-Control group, two-way ANOVA with repeated measures. **(E)** Typical EEGs and power spectrogram recorded from the hippocampus during seizures; the black bar indicates kindling stimulation artifact.

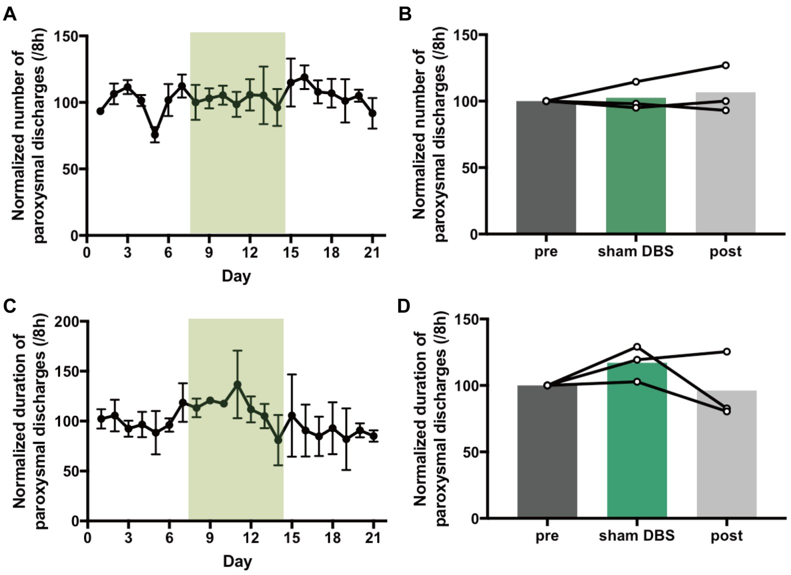

Figure S3 The effect of sham DBS in the MS on seizure activities in KA-induced chronic epileptic mice. (A-D) The effects of the sham DBS in the MS on the normalized number of paroxysmal discharges **(A, B)** and the normalized duration of paroxysmal discharges **(C, D)** in KA-induced chronic epilepsy model. To minimize individual variation, the data for each mouse (n=3) was normalized to the average value of the 7-days pre-sham DBS.
